# Non-hormonal systemic therapy in men with hormone-refractory prostate cancer and metastases: a systematic review from the Cancer Care Ontario Program in Evidence-based Care's Genitourinary Cancer Disease Site Group

**DOI:** 10.1186/1471-2407-6-112

**Published:** 2006-05-02

**Authors:** Eric Winquist, Tricia Waldron, Scott Berry, D Scott Ernst, Sébastien Hotte, Himu Lukka

**Affiliations:** 1 Department of Medical Oncology, London Health Sciences Centre, London, Ontario, Canada; 2 Department of Clinical Epidemiology and Biostatistics, Cancer Care Ontario Program in Evidence-based Care, McMaster University, Hamilton, Ontario, Canada; 3 Department of Medical Oncology, Toronto-Sunnybrook Regional Cancer Centre, Toronto, Ontario, Canada; 4 Division of Hematology/Oncology, Miller School of Medicine, University of Miami, Miami, Florida, USA; 5 Department of Medical Oncology, Juravinski Cancer Centre, Hamilton, Ontario, Canada; 6 Department of Radiation Oncology, Juravinski Cancer Centre, Hamilton, Ontario, Canada

## Abstract

**Background:**

Prostate cancer that has recurred after local therapy or disseminated distantly is usually treated with androgen deprivation therapy; however, most men will eventually experience disease progression within 12 to 20 months. New data emerging from randomized controlled trials (RCTs) of chemotherapy provided the impetus for a systematic review addressing the following question: which non-hormonal systemic therapies are most beneficial for the treatment of men with hormone-refractory prostate cancer (HRPC) and clinical evidence of metastases?

**Methods:**

A systematic review was performed to identify RCTs or meta-analyses examining first-line non-hormonal systemic (cytotoxic and non-cytotoxic) therapy in patients with HRPC and metastases that reported at least one of the following endpoints: overall survival, disease control, palliative response, quality of life, and toxicity. Excluded were RCTs of second-line hormonal therapies, bisphosphonates or radiopharmaceuticals, or randomized fewer than 50 patients per trial arm. MEDLINE, EMBASE, the Cochrane Library, and the conference proceedings of the American Society of Clinical Oncology were searched for relevant trials. Citations were screened for eligibility by four reviewers and discrepancies were handled by consensus.

**Results:**

Of the 80 RCTs identified, 27 met the eligibility criteria. Two recent, large trials reported improved overall survival with docetaxel-based chemotherapy compared to mitoxantrone-prednisone. Improved progression-free survival and rates of palliative and objective response were also observed. Compared with mitoxantrone, docetaxel treatment was associated with more frequent mild toxicities, similar rates of serious toxicities, and better quality of life. More frequent serious toxicities were observed when docetaxel was combined with estramustine. Three trials reported improved time-to-disease progression, palliative response, and/or quality of life with mitoxatrone plus corticosteroid compared with corticosteroid alone. Single trials reported improved disease control with estramustine-vinblastine, vinorelbine-hydrocortisone, and suramin-hydrocortisone compared to controls. Trials of non-cytotoxic agents have reported equivocal results.

**Conclusion:**

Docetaxel-based chemotherapy modestly improves survival and provides palliation for men with HRPC and metastases. Other than androgen deprivation therapy, this is the only other therapy to have demonstrated improved overall survival in prostate cancer in RCTs. Further investigations to identify more effective therapies for HRPC including the use of systemic therapies earlier in the natural history of prostate cancer are warranted.

## Background

Prostate cancer is the most frequently diagnosed cancer in North America, and the third most common cause of cancer death in men [1]. Men with prostate cancer that has recurred after local therapy or disseminated distantly usually respond to androgen deprivation therapy (ADT). However, most patients eventually experience disease progression within a median of 12 to 20 months [2]. Androgen independence is defined as disease progression despite effective ADT, typically first identified by a rise in serum prostatic-specific antigen (PSA) levels. Hormone-refractory prostate cancer (HRPC) arises when disease progression continues despite secondary hormonal maneuvers and antiandrogen withdrawal (AAWD). The prognosis of HRPC is associated with performance status, the presence of bone pain, extent of disease on bone scan, and serum alkaline phosphatase levels [3]. Bone metastases will occur in 90% of men with HRPC and can produce significant morbidity including pain, pathologic fractures, spinal cord compression, and bone marrow failure [4]. Paraneoplastic effects are also common, including anemia, weight loss, fatigue, hypercoagulability, and increased susceptibility to infection. Thus, HRPC presents a spectrum of disease ranging from patients without metastases or symptoms with rising PSA levels despite ADT, to patients with metastases and significant debilitation due to cancer symptoms. Historically, clinical management has been primarily palliative with a focus on expectant management when possible and palliative interventions such as radiotherapy, radioisotopes, and chemotherapy when necessary [5]. New data emerging from large clinical trials of chemotherapy provided the impetus for this systematic review of the value of chemotherapy and other non-hormonal agents in HRPC.

## Methods

### Development of systematic review

The Genitourinary Cancer Disease Site Group of the Cancer Care Ontario Program in Evidence-Based Care (CCOPEBC) has developed an evidence-based clinical practice guideline on the use of non-hormonal systemic therapy for HRPC using the methodology outlined in the practice guideline development cycle by Browman et al [6]. The guideline was derived from a systematic review and input from practitioners in Ontario, Canada. This report describes the systematic review portion of the guideline, which addressed the following question: which non-hormonal systemic therapies are most beneficial for the treatment of men with HRPC and clinical evidence of metastases?

### Inclusion criteria

Articles were selected for inclusion in the systematic review if they were randomized controlled trials (RCTs) or meta-analyses of RCTs studying a first-line non-hormonal systemic (cytotoxic and non-cytotoxic) therapy in patients with HRPC and metastases that reported on at least one of the following outcomes: overall survival, disease control (i.e., progression-free survival [PFS], time-to-progression [TTP], time-to-treatment failure, objective tumor response, and PSA response), palliative or symptomatic response, quality of life (QoL), and/or toxicity. Previous systematic reviews or evidence-based guidelines that addressed non-hormonal systemic therapy in HRPC were also eligible for inclusion.

### Exclusion criteria

Articles were excluded from the systematic review if they studied second-line hormonal therapies, bisphosphonates or radiopharmaceuticals, or randomized fewer than 50 patients per trial arm. When formulating the protocol for this review, the authors were aware of dozens of small randomized trials comparing the activity of various drugs in HRPC. Sometimes these were identified explicitly as phase II trials, but often they were not. Although valuable for identifying potential anti-tumor activity, such trials are by definition underpowered to address the patient outcomes of interest to this review, and often did not report these. Although these limitations could be overcome by statistical pooling, it is also recognized that such RCTs are associated with more variability and are more likely to be reported and published if positive [7]. Theoretically, positive results from such trials require subsequent confirmation by larger pragmatic RCTs, but this does not always occur. After considering the outcomes of interest for this review, a minimum sample size of 50 randomized patients per trial arm was chosen, in order to be as inclusive as possible while still minimizing inclusion of randomized phase II trials. This sample size was based on a requirement of at least 80% power for an RCT to reliably detect the difference between an endpoint response rate of 10% versus 30% with one-tailed α = 0.05. Reliable assessment of outcomes such as disease-free and overall survival would require even larger sample sizes. RCTs with lesser discriminating ability were considered underpowered and potentially misleading with regard to those endpoints.

### Literature search strategy

The MEDLINE (1966 through March 2004), EMBASE (1980 through 2004, week 10), and Cochrane Library (2003, Issue 4) databases (Central Register of Controlled Trials [CCTR] and Database of Systematic Reviews [DSR]), and the conference proceedings of the American Society of Clinical Oncology (1999 through 2004) were searched for abstracts of relevant trials. MEDLINE was searched using the following medical subject headings (MeSH): "prostatic neoplasms", "drug therapy", "antineoplastic agents", and "drug therapy, combination"; and EMBASE was searched using the following Excerpta Medica tree terms: "prostate tumor", "prostate cancer", "drug therapy", "antineoplastic agent", "drug combination", and "combination chemotherapy". In each database, those subject headings were combined with disease and treatment-specific text words (e.g., "prostate cancer", "prostate tumor", "prostate carcinoma", and "chemotherapy"). Those terms were then combined with the search terms for the following publication types and study designs: practice guidelines, systematic reviews, meta-analyses, reviews, randomized controlled trials, and controlled clinical trials. The CCTR and DSR databases were searched using a combination of the aforementioned MeSH and keywords. The reference lists from eligible articles were searched for additional trials, as were the reference lists from relevant review papers.

### Methods of review

Citations identified by the literature search strategy were screened for eligibility by four of the authors (EW, DSE, SB, SH) and discrepancies were handled by consensus. Data pertaining to trial design, participants, interventions, and outcomes were extracted from each eligible trial by one reviewer (TW) and were audited by a second reviewer (EW) independently. Information indicative of trial quality, including methods of randomization, absence or degree of blinding, completeness of patient follow-up, and whether statistical analyses were performed by intent-to-treat were also extracted from each trial report. The quality parameters assessed on each trial were used to identify any trials with serious flaws in trial methodology that could lead to potentially misleading results.

### Synthesis of the evidence

This review is based on data provided by published reports. When pooling of data is to be undertaken and individual patient data are not available, methods are available for generating summary statistics from published data. However, statistical pooling (meta-analysis) of data from RCTs is only possible if control arms are similar and is only valid if those data are statistically homogeneous. Reports of RCTs studying systemic therapies in HRPC date back 30 years and have studied heterogeneous patient populations, interventions, and outcomes. Numerous drug interventions have been tested, including a variety of single-agent and combination chemotherapy regimens including estramustine phosphate (EMP), and non-cytotoxic drugs such as liarazole, suramin, and atrasentan. What constitutes standard therapy in control arms has been controversial and has included placebo, corticosteroids, EMP, and cytotoxics. On the basis of those observations, large-scale quantitative pooling of RCT data was considered neither possible nor appropriate, and so an interpretive summary of the data was planned. The natural history and management of HRPC has changed in the last three decades; therefore, more contemporary studies were emphasized in the interpretive summary to provide clinicians with the evidence most relevant to current practice. Furthermore, more emphasis on the results of RCTs demonstrating internally consistent benefits in survival, palliation, and quality of life outcomes was planned.

## Results

### Literature search results

The literature search identified 80 unique RCTs that compared non-hormonal systemic treatments in HRPC. Fifty-three of those trials randomized less than 50 patients per arm and were considered ineligible [8-60] (see Additional file 1). Of the 27 eligible trials, 22 were published as full reports [61-83], and five were available only in abstract or poster presentation form but provided adequate data on at least one outcome of interest [84-88]. There were 21 two-arm and six three-arm trials, and two trials were labeled as randomized phase II reports [79,84]. No published systematic reviews or evidence-based guidelines were identified. No trials were excluded due to their quality.

The 27 RCTs that form the basis of this review were published between 1979 and 2004. A total of 7489 eligible men were randomized, ranging from 102 to 1006 per RCT. Six trials were placebo-controlled [65,79,82,85-87], and four of those were also double-blinded [65,79,82,85]. Twelve trials described the methods used to randomize patients [61-65,70-73,79,83,86], and 21 reported that treatment arms were balanced for important baseline prognostic factors [61-67,69-73,75-79,81-83,87]. Twelve trials performed statistical analyses according to intent-to-treat [62-64,70,71,74,79,81-83,85,86]. A minority of trials reported whether patients underwent AAWD [61-64,69-71,79,81,83] and continued to receive ADT during the study period [61-64,69-71,81-83]. Twenty trials studied cytotoxic [61-78,84,88] and seven studied non-cytotoxic drug interventions [79-83,85-87]. Two cytotoxic trials studied agents belonging to multiple drug classes [75,88]. For clarity, the cytotoxic trials have been organized according to the drug class tested by the trial: antimicrotubule-based regimens (nine trials), anthracendedione/anthracycline-based regimens (nine trials), and other chemotherapy (four trials).

### Antimicrotubule-based chemotherapy

#### Docetaxel

Docetaxel induces polymerization of microtubules and phosphorylation of bcl-2 protein. Two large RCTs comparing docetaxel-based chemotherapy to mitoxantrone-prednisone have recently been published (Table 1). Tannock et al [64] randomized 1006 patients to one of three treatment arms: docetaxel (75 mg/m^2 ^intravenously [iv] every [q] three weeks), docetaxel (30 mg/m^2 ^iv five-times weekly for five of six weeks), or control therapy with mitoxantrone. Patients in all three arms also received prednisone (5 mg orally [po] twice daily). Petrylak et al [63] reported on 666 eligible patients randomized to docetaxel and EMP or mitoxantrone-prednisone. In addition to dexamethasone premedication, patients in the docetaxel arm also received warfarin and acetylsalicylic acid (ASA) as thrombosis prophylaxis during the course of the trial. Men in both trials had clinical evidence of metastases with or without symptoms and had undergone AAWD. Overall survival was the primary endpoint in both trials.

Tannock et al [64] reported improved survival with docetaxel-prednisone (q third week) compared with mitoxantrone-prednisone (median survival, 18.9 versus 16.5 months; hazard ratio [HR] = 0.76 [95% confidence interval (CI), 0.62–0.94], two-sided p = 0.009) (Table 2). No overall survival benefit was observed with docetaxel-prednisone given on a weekly schedule (HR = 0.91, [95% CI, 0.75–1.11], two-sided p = 0.36). Petrylak et al [63] reported longer survival time with docetaxel-EMP combination chemotherapy compared with mitoxantrone-prednisone (median survival, 17.5 versus 15.6 months; HR = 0.80 [95% CI, 0.67–0.97], two-sided p = 0.02) (Table 2). This trial also reported a median progression-free interval of 6.3 versus 3.2 months (HR = 0.73 [95% CI, 0.63–0.86], two-sided p < 0.0001) favoring docetaxel-EMP compared with mitoxantrone-prednisone.

Pain response was assessed in both trials. Significantly more patients treated with docetaxel-prednisone (q third week) achieved a pain response compared with patients receiving mitoxantrone-prednisone (35% versus 22%, p = 0.01) [64]. A trend towards improved pain response was observed with weekly docetaxel-prednisone versus mitoxantrone-prednisone (31% versus 22%, p = 0.08). QoL response defined as a sustained 16-point or greater improvement from baseline on two consecutive measurements was higher with docetaxel given every three weeks (22% versus 13%, p = 0.009) or weekly (23% versus 13%, p = 0.005) compared with mitoxantrone. Petrylak et al [63] reported no difference in patient reported pain relief between arms in their trial and did not assess QoL.

In both trials, PSA response rates were also statistically significantly higher with docetaxel compared to mitoxantrone (Table 3). Twenty-seven per cent (n = 412) [64] and 29% (n = 196) [63] of patients in the two trials had measurable disease. Objective response rates for docetaxel-prednisone (q three weeks) and mitoxantrone-prednisone were 12% versus 7%, respectively (Table 3). Petrylak et al [63] reported objective response rates of 17% and 11% favoring docetaxel-EMP compared with mitoxantrone-prednisone. The differences in objective response rates between arms were not statistically significant in either trial.

More grade 3–4 neutropenia (32% and 22% versus 1.5%) and neutropenic infection (3% and 0.9% versus 0%) were observed with docetaxel-prednisone (q third week) compared with mitoxantrone-prednisone and docetaxel-prednisone given weekly, respectively [64]. However, only two septic deaths occurred, one each in the mitoxantrone and weekly docetaxel arms. Grade 3–4 non-hematological toxicities were infrequent and similar in the docetaxel and mitoxantrone arms. Mild to moderate alopecia, fatigue, diarrhea, nail changes, stomatitis, peripheral edema, anorexia, and dyspnea were more common with docetaxel. More grade 3–4 toxicity (53% versus 33%) was associated with docetaxel-EMP compared with mitoxantrone-prednisone, primarily due to higher rates of gastrointestinal and cardiovascular events [63]. The protocol was amended to add oral coumadin (2 mg daily) and oral ASA (325 mg daily) to the docetaxel arm, but post hoc analysis suggested prophylactic anticoagulation had little effect on the rate of thromboembolic events. Docetaxel-EMP was also associated with statistically significantly higher rates of metabolic disturbances (6% versus 1%) and neurologic events (7% versus 2%) compared to mitoxantrone-prednisone. Eight (2%) versus four (1%) toxic deaths occurred in the docetaxel-EMP and mitoxantrone arms, respectively.

#### Estramustine

EMP is a nor-nitrogen mustard carbamate derivative of estradiol-17β-phosphate with estrogenic and antimicrotuble effects. It is unclear how much of this agent's activity in HRPC is due to its hormonal versus its cytotoxic effects. Six RCTs directly examined the efficacy of EMP in HRPC (Table 1). Three studied EMP either by comparing it to a placebo or an oral antiandrogen [65-67], and three added EMP to a cytotoxic agent and compared this combination to the cytotoxic agent alone [61,68,84]. One other large RCT comparing docetaxel-EMP to mitoxantrone-prednisone could be considered to indirectly address the value of EMP (see *Docetaxel *above) [63].

All six trials reported on overall survival, but none detected improvements with EMP (Table 2). Five trials reported TTP or PFS results; of those, one trial comparing EMP-vinblastine to vinblastine alone reported longer TTP with the combination (median, 3.7 versus 2.2 months, one-sided p < 0.0004) [61]. EMP was not associated with improved pain, performance status, or subjective response rate in two trials reporting those data [65,68]. Hudes et al [61] reported improved pain frequency with EMP; however, less than 50% of patients with pain completed pain questionnaires. QoL data were also collected in that trial but did not allow comparative assessment. The three RCTs reporting on PSA response showed higher PSA response rates with EMP [61,65,84] (Table 3). Three RCTs reported objective response rates [61,67,68] and none showed improved tumor response with EMP (Table 3).

EMP was generally associated with clinically significant higher rates and severity of gastrointestinal toxicity (including nausea and vomiting, diarrhea, and dyspepsia), breast tenderness/gynecomastia, leg edema, thrombosis, and cardiovascular deaths [61,65-67]. The addition of EMP to chemotherapy was also associated with a reduced incidence and severity of neutropenia [68].

#### Vinorelbine

Vinorelbine is a semi-synthetic vinca alkaloid with single-agent activity in HRPC. Abratt et al [62] randomized 414 men treated with hydrocortisone with or without aminoglutethimide to vinorelbine or no chemotherapy (Table 1). The primary endpoint of the trial was PFS. A longer progression-free interval was reported with vinorelbine after adjustment for predetermined prognostic factors (median, 3.7 versus 2.8 months; HR = 0.71, unadjusted two-sided p = 0.055, adjusted two-sided p = 0.005). No difference in overall survival was detected (Table 2). Thirty-four percent of patients (n = 142) had measurable disease and response rates of 5.9% (partial only) and 0% were reported favoring vinorelbine (p-value not reported) (Table 3). PSA response rates (Table 3) and clinical benefit response (defined as improved pain, analgesic score, or performance status for greater than nine weeks) were higher with vinorelbine compared with the control arm (30.6% versus 19.2%, p = 0.008). QoL data were collected in the trial but were limited due to poor patient compliance and use of a general rather than a specific prostate cancer QoL instrument; and showed no benefit with vinorelbine on either global QoL or functional subscales. More frequent severe neutropenia (26%), neutropenic infection (3%), anemia (6.5%) and constipation (3%) were observed with the addition of vinorelbine to hydrocortisone with or without aminoglutethimide.

### Anthracenedione/anthracycline-based chemotherapy

#### Mitoxantrone

Mitoxantrone is an anthracenedione drug with mechanisms of activity similar to anthracyclines and a modest toxicity profile. Three RCTs compared mitoxantrone combined with low-dose corticosteroid to the same low-dose corticosteroid alone without placebo [69-71] (Table 4). In the largest trial, Kantoff et al [70] randomized 242 patients with metastatic HRPC who had undergone AAWD to either mitoxantrone plus hydrocortisone or hydrocortisone alone. The primary endpoint of the trial was overall survival. Tannock et al [71] compared mitoxantone plus prednisone to prednisone alone in 161 men with HRPC symptomatic with pain. Pain relief was the primary endpoint of that trial, defined by patient self-reported pain intensity and analgesic use as recorded in an analgesic diary. The analysis of overall survival was confounded by crossover to the mitoxantrone arm at the time of cancer progression in this trial. Berry et al [69] evaluated the same treatment regimens as Tannock et al in 120 men with asymptomatic HRPC using TTP as the primary endpoint.

All three RCTs reported overall survival results but none detected an improvement due to mitoxantrone (Table 5). Berry et al [69] and Kantoff et al [70] both reported longer median TTP with mitoxantrone versus control (8.1 versus 4.1 months [p = 0.018], and 3.7 versus 2.3 months [p = 0.02], respectively) (Table 5). Objective response rates were reported in two trials [69,70] without differences observed (Table 6). All three trials reported PSA response rates, which were significantly higher with mitoxantrone in one trial [69] (Table 6).

In the only trial evaluating pain, Tannock et al [71] rigorously assessed palliative response through self-reported pain scores and analgesic consumption. In the mitoxantrone-prednisone treatment arm, 29% of patients had a two-point reduction in pain intensity (or complete elimination of pain) on the six-point McGill-Melzack Pain Questionnaire, maintained for three weeks apart without an increase in analgesic use, compared with 12% of patients treated with prednisone alone (p = 0.01). The median duration of pain response was 43 versus 18 weeks favoring mitoxantrone (p < 0.0001). An additional seven patients in each arm had a decrease of ≥ 50% in analgesic score without an increase in pain; thus, 38% of patients treated with mitoxantrone-prednisone had palliative benefit compared with 21% with prednisone alone (p = 0.025).

Two trials reported QoL data [70,71]. Tannock et al [71] reported improved QoL with mitoxantrone-prednisone over prednisone alone in domains related to pain, physical activity or function, constipation, and mood with the Prostate Cancer Specific QoL Instrument and the European Organization for Research and Treatment of Cancer Quality of Life Core Questionnaire (EORTC QLQ-C30). Patients meeting the criteria for palliative response had improvements in most QoL domains, including overall well-being. Kantoff et al [70] also reported improved QoL favoring mitoxantrone in the Functional Living Index: Cancer (FLIC) emotional state and family disruption subscales.

All three trials reported toxicity. Grade 3–4 neutropenia occurred in 45% of cycles, and 63% and 48% of patients, respectively [69-71]. Neutropenic sepsis occurred in 6.9% and 2% of patients [69,71]. Severe symptomatic non-hematological toxicities were rare; for example, severe nausea and vomiting occurred in 0.5% of cycles [71]. Cardiac dysfunction, either symptomatic or detected by reduced LVEF, was observed in 3.8% and 5% of patients [69,71]. No toxic deaths were observed with mitoxantrone in any of those trials.

#### Doxorubicin and epirubicin

Anthracyclines are believed to exert their cytotoxic effects primarily through the inhibition of topoisomerase-II activity. Six RCTs examined anthracycline combinations [72-76,88] (Table 4). Four performed in the pre-PSA era compared doxorubicin-based cytotoxic chemotherapy regimens to non-doxorubicin-based regimens or single agents [74-76] or compared combined versus sequential 5-flourouracil-doxorubicin-mitomycin-C (FAM) [73]. Two trials compared anthracyclines to EMP [72,88]. The primary endpoints of those trials were tumor response and survival [73-76] and TTP [72].

Only one of five RCTs reporting overall survival data reported improved survival with chemotherapy [72-76,88] (Table 5). Laurie et al [73] randomized 142 patients to either combination chemotherapy with FAM or sequential chemotherapy with the same drugs (mitomycin C followed by doxorubicin followed by 5-fluorouracil). Although response rates were similar between the two arms and hematological toxicity was greater with FAM, overall survival favored the combined FAM regimen (median survival, 8.7 versus 7.1 months, p = 0.025). Three trials provided comparative data on disease progression [72,74,75]; two reported on TTP [72,74] and one reported on PFS [75] (Table 5). Improved TTP was detected with epirubicin and MPA compared with EMP (median, 7.6 versus 4.3 months, p = 0.013) [72].

Five trials reported on objective tumor response [73-76,88] (Tables 6). Only two of the five trials provided statistical comparisons of those data; one detected no difference and the other reported higher response rates with combined doxorubicin-cyclophosphamide chemotherapy (32%) versus hydroxyurea (4%) that was of borderline statistical significance (p = 0.05) [76]. Two of the trials assessed a pain or palliative endpoint [76,88]. Stephens et al [76] reported that symptomatic response rate (a composite endpoint comprising of worsening symptoms and analgesic use) was higher with doxorubicin plus cyclophosphamide compared with hydroxyurea (26% versus 13%, p = 0.048) but the duration of that response was not significantly different between the two groups (p = 0.62). The second trial [88] reported comparable rates of pain relief (undefined) among patients treated with epirubicin (49%), mitomycin C (48%), and EMP (42%). None of the six trials reported QoL data.

#### Other cytotoxic agents

Four trials studied other chemotherapy agents [75,77,78,88] (Table 7). The National Prostatic Cancer Project (NPCP) randomized 189 men with clinically progressing HRPC to either single-agent cisplatin, methotrexate, or EMP [78]. In a successor trial, 180 patients were randomized to either single-agent methotrexate, combination cyclophosphamide-5-fluorouracil-cisplatin, or cyclophosphamide-doxorubicin [75]. Objective response by NPCP criteria was the primary endpoint of both trials. Newling et al [77] compared mitomycin C with EMP in 171 randomized patients with TTP and overall survival as primary endpoints. All three trials were completed during the pre-PSA era. Weissbach et al [88] randomized 175 patients to mitomycin C, epirubicin, or EMP.

All four trials reported data on overall survival [75,77,78,88], but none reported differences between trial arms. Two trials reported on disease progression [75,77] and no differences were detected. Improved time-to-treatment failure was reported with mitomycin C compared with epirubicin (p = 0.039) and EMP (p = 0.037) [88]. Trials reporting symptomatic or pain response identified no differences [78]. Three trials [75,78,88] reported tumor response data and no differences were observed. One trial collected QoL data, but it was of limited value due to missing data [77].

#### Non-cytotoxic agents

Five non-cytotoxic agents, including liarozole, suramin, atrasentan, prinomastat, and APC8015 have been investigated in RCTs in HRPC (Table 8) [79-83,85-87]. Liarozole is thought able to promote the differentiation of malignant cells by increasing intracellular levels of retinoic acid. Debruyne et al [83] randomized 321 patients to either liarozole or cyproterone acetate. Suramin is a highly charged polysulfonated napthylurea with antineoplastic activity of uncertain mechanisms and adrenolytic effects. Two recent, large RCTs have studied suramin in men with HRPC. In a placebo-controlled trial, Small et al [82] studied the effects of suramin plus hydrocortisone to hydrocortisone alone in men with HRPC and pain requiring opioid analgesics. The primary endpoint was pain response. A subsequent RCT compared three different doses of suramin and evaluated PSA response as the primary endpoint [81]. Atrasentan is an orally bioavailable endothelin A antagonist. Two large RCTs have studied atrasentan in comparison to placebo in men with HRPC [79,85]. The matrix metalloprotease inhibitor prinomastat has been combined with mitoxantrone-prednisone and compared with placebo [87]. APC8015 is a cellular therapy consisting of autologous peripheral blood mononuclear cells enriched for dendritic cells and pulsed with a prostatic acid phosphatase-GM-CSF construct. APC8015 has also been compared with placebo in men with HRPC [86].

Four of the seven trials of non-cytotoxic agents reported overall survival results, and none reported differences in overall survival between treatment arms (Table 9) [80-83,87]. Reduced mortality was reported when liarozole was compared with cyproterone acetate after an adjustment for prognostic factors by Cox multivariate regression analysis (HR = 0.74 [95% CI, 0.56–0.99], p = 0.039) [83]. All eight trials reported on a disease-progression outcome; four trials reported TTP [79,82,85,86], three reported on PFS [81,83,87] and one reported failure-free survival data [80] (Table 9). Of those trials, two detected statistically significant differences favoring the experimental treatment [80,82]. TTP was improved with suramin-hydrocortisone compared with placebo-hydrocortisone (relative risk = 1.51 [95% CI, 1.22–1.85], two-sided p = 0.0003) [82], but was not affected by suramin schedule in another trial [81]. Tumor response data were reported in three trials [80-82] (Table 10), of which only one detected significant differences between trial arms [80]. PSA response rates were reported in seven trials [79,81-83,85-87]; four of those detected statistically significantly higher response rates with the experimental therapy [79,82,83,85] (Table 10).

Three trials reported pain or symptomatic response data [80,82,83,86]. The mean best change in pain and analgesic use score compared with baseline was improved with liarozole compared with cyproterone acetate (mean reduction 0.4 versus 0.2, p = 0.026) [83]. Small et al [82] assessed palliative response to suramin with self-reported pain scores and opioid analgesic use using two methods. For the first method, average worst pain scores (during the previous 24 hours) measured with the Brief Pain Inventory (BPI) and opioid analgesic consumption were assessed in each treatment group and compared with baseline at six weeks and at the end of treatment. Suramin was superior to placebo for pain reduction at both six weeks and the end of study; no statistically significant differences in narcotic analgesic consumption were observed. For the second method, pain response was measured and defined either by a three-point reduction (or complete elimination) of worst pain on the BPI (maintained for at least three weeks) with a <16% increase in opioid analgesic use or by a ≥ 33% (minimum 5 mg) reduction in opioid analgesic use with a two-point or less increase in pain. More patients in the suramin group had pain response compared with placebo (43% versus 28%, p = 0.001), and the duration of pain response was significantly higher (median 240 versus 69 days, two-sided p = 0.0027). Performance status measured by the Revised Rand Functional Limitations Scale was not improved with suramin compared with placebo. Three trials assessed QoL outcomes [79,82,83] and none reported differences.

Liarozole was associated with increased rates of skin toxicity, nausea and vomiting, and fatigue [83]. In comparison to placebo, suramin was associated with more frequent mild to moderate rash (57% versus 13%) and severe edema and anemia (both <5%) [82]. Higher rates of severe toxicities were seen with high-dose suramin, including neutropenia, anorexia, cardiac dysrhythmias, and neuromotor toxicity [81]. Prinomastat was associated with increased rates of mild to moderate musculoskeletal effects including arthralgia, joint stiffness and swelling, and, rarely, contracture compared with placebo [87]. Atrasentan was associated with increased rates of mild to moderate peripheral edema, rhinitis, headache, hypotension, anemia, and weight gain compared with placebo [79].

## Discussion

The diagnosis and clinical management of HRPC has undergone radical changes over the past decade, along with the design and methodology of clinical trials. A "stage migration" has occurred due to the ability of PSA testing to detect biochemical evidence of androgen independence before other clinical symptoms or signs become apparent. In the pre-PSA era, men with HRPC enrolled in RCTs were often symptomatic and extensively pre-treated with palliative radiotherapy. Androgen levels influence tumor growth in HRPC, and differences in or lack of control of ADT used in trial subjects might affect outcomes. For example, luteinizing hormone-releasing hormone (LHRH)-agonist use has become much more prevalent over the past decade, replacing estrogens and reducing the use of bilateral orchiectomy. As well, the AAWD syndrome has been identified as a potential confounder of clinical and biochemical response in HRPC [89]. Withdrawal of oral antiandrogens and maintenance of ADT were required for entry onto the largest clinical trials [63,64,70,71] discussed in this review. Generalizability, changes in use of androgen deprivation therapy, and the validity of trial endpoints need to be considered in the interpretation and weighting of the evidence provided by clinical trials in HRPC.

Typically, men with HRPC have skeletal metastases that cannot be conventionally assessed for objective response to anticancer therapy. As a result, drug trials have used a number of different primary endpoints. The identification of benefits from non-hormonal drug therapy only became clear over the past 15 years with the emergence first of validated symptom and quality of life instruments, then the availability of PSA as a tumor marker, and finally the ability to conduct randomized trials large enough to adequately assess survival benefits. As the purpose of this review was to inform clinical practice, endpoints unequivocally associated with patient benefit or harm were emphasized, as were RCTs of sufficient power to assess these.

Early RCTs studying several single-agent and combination chemotherapy regimens compared with other single-agent chemotherapy controls showed evidence of modest anti-tumor activity generally accompanied by increased toxicity. Interpretation of the results of these trials was limited by their sample sizes and lack of validated psychometric tools to ascertain palliative treatment benefits. Tannock et al [71] established mitoxantrone-prednisone as a standard palliative therapy for men with HRPC symptomatic with pain. Two trials [69,70] subsequently confirmed that mitoxantrone also improved TTP compared to initial corticosteroid therapy alone. The lack of toxicity in those trials was notable, with no toxic deaths and few serious hematological and non-hematological side effects. Cardiomyopathy was observed in ≥ 5% of patients in those trials. Improved PFS was also associated with vinorelbine, along with a modest benefit in clinical benefit response [65]. A number of agents with novel mechanisms of anti-tumor activity have been studied in HRPC; and although activity and some benefits have been observed, data from RCTs has not yet established any of these agents as standard therapeutic options for HRPC. The strategy of adding EMP to chemotherapy has also been explored, and PSA response and disease control appear modestly improved [64,68,87]. However, overall survival is not clearly increased, and EMP is associated with adverse effects that include thrombosis and cardiovascular toxicity [61,63,65,84].

Improvement in overall survival has been reported with docetaxel given every three weeks in comparison with mitoxantrone-prednisone in two large, well-conducted RCTs [63,64]. Docetaxel-prednisone given on a weekly schedule was not clearly associated with improved overall survival [64]. These trials also reported evidence of improved PFS, palliative response, and/or objective response with all the docetaxel regimens studied. Docetaxel-prednisone (without estramustine) was associated with more frequent mild toxicities, similar rates of serious toxicities, and better QoL than mitoxantrone-prednisone. Based on this evidence, docetaxel given every third week with either daily prednisone or EMP appears to be the most effective drug treatment tested in RCTs for men with HRPC and metastases; however, indirect comparison suggests the latter regimen may be associated with more frequent and severe toxicities than the former.

## Conclusion

Docetaxel-based chemotherapy given every three weeks was the only treatment that demonstrated an overall survival benefit in men with HRPC. Most men receiving docetaxel had metastases, so the timing of docetaxel therapy in men without metastases should not only be carefully considered but also studied further in clinical trials. Expectant management, trials of secondary hormonal manipulations, and/or participation in clinical trials of investigational agents before chemotherapy are also reasonable alternatives for many men with HRPC on an individualized basis. In the largest randomized trials, men continued on gonadal androgen suppression and discontinued the use of oral antiandrogens, and these maneuvers are recommended for men planned to receive chemotherapy. Use of EMP in combination with other cytotoxic agents is probably not worthwhile due to the increased risk of clinically important toxicities without clear evidence of improved survival or palliation. Mitoxantrone-prednisone and weekly docetaxel-prednisone are associated with symptom palliation and improved disease control without improved overall survival, and can be considered clinical alternatives to docetaxel given every three weeks.

## Competing interests

EW has received honoraria from Sanofi-Avenitis. TW has no competing interests. SB has served as a consultant and received honoraria and research funding from Aventis. DSE has served as a consultant and received honoraria from Novartis, and has received honoraria and research funding from Aventis. SH has no competing interests. HL has received honoraria from Aventis.

## Authors' contributions

EW was the lead author responsible for the conception and design of the systematic review, screening of citations, data auditing, analysis and interpretation, and drafting and editing of the manuscript. TW conducted literature searches, data extraction, and drafted and edited the manuscript. SB, DSE, and SH also screened citations; and SB, DSE, SH and HL reviewed all drafts of the manuscript and made major contributions to the interpretation of the evidence and manuscript revisions. All authors read and approved the final manuscript.

## Pre-publication history

The pre-publication history for this paper can be accessed here:



## Supplementary Material

Additional file 1 **Appendix of ineligible randomized trials**The file is a word document and describes the randomized trials that were excluded from the systematic review because they did not meet the eligibility criteria. Click here for file

## Figures and Tables

**Table 1:** Antimicrotubule trials.

**Trial**	**N randomized/evaluable**	**Treatment arms (dose) and schedule**	**Duration**
Tannock, 2004 [64]	1006/1006	docetaxel (75 mg/m^2^) iv q 3 wks prednisone (5 mg) po twice daily dexamethasone (8 mg) at 12, 3, and 1 hr(s) prior to infusion	10 cycles
		docetaxel (30 mg/m^2^) iv q wk for 5 wks prednisone (5 mg) po twice daily dexamethasone (8 mg) at 1 hr prior to infusion	5 cycles
		mitoxantrone (12 mg/m^2^) iv q 3 wks prednisone (5 mg) po twice daily	10 cycles
Petrylak, 2004 [63]	770/666	docetaxel (60 mg/m^2^)* iv q 3 wks EMP (280 mg) po thrice daily q 3 wks dexamethasone (20 mg) po thrice daily q 3 wks	12 cycles
		mitoxantrone (12 mg/m^2^)* iv q 3 wks prednisone (5 mg) po twice daily q 3 wks	
Abratt, 2003 [62]	451/414	vinorelbine (30 mg/m^2^) iv q 3 wks hydrocortisone (40 mg) ± AGM (1000 mg)† daily	to progression
		hydrocortisone (40 mg) ± AGM (1000 mg)† daily	
Berry, 2001 [84]	166/NR	paclitaxel (100 mg/m^2^) iv q 4 wks EMP (280 mg) po thrice daily	NR
		paclitaxel (100 mg/m^2^) iv q 4 wks	
Hudes, 1999 [61]	201/193	vinblastine (4 mg/m^2^) iv q wk for 6 of 8 wks EMP (600 mg/m^2^) po daily (2 or 3 divided doses)	to progression
		vinblastine (4 mg/m^2^) iv q wk for 6 of 8 wks	
Iversen, 1997 [65]	131/129	EMP (560 mg) po daily (2 divided doses)	as long as tolerated by patient
		placebo po daily	
Johansson, 1991 [66]	105/102	MPA (1000 mg) im daily (d1-15), then im weekly	to progression
		EMP (280 mg) po twice daily	
De Kernion, 1988 [67]	220/203	EMP (600 mg/m^2^) po (3 divided doses)	NR
		flutamide (0.25 gm) po thrice daily	
Murphy, 1979 [68]	135/116	EMP (600 mg/m^2^) po daily (3 divided doses) prednimustine (30 mg) po daily (3 divided doses) q wk	to progression
		prednimustine (30 mg) po daily (3 divided doses) q wk	

*Docetaxel could be increased to 70 mg/m^2 ^and mitoxantrone could be increased to 14 mg/m^2 ^if no grade 3 or 4 toxicities were observed in cycle 1; †decision to use AGM was at the discretion of participating centers.

Abbreviations: AGM – aminoglutethimide; d – day; EMP – estramustine phosphate; hr – hour; im – intra muscular; iv – intravenous; mg – milligrams; MPA – medroxyprogesterone acetate; m^2 ^- meters squared; N – number; NR – not reported; po – per oral; q – every; wk(s) – week (s).

**Table 2:** Antimicrotubule trials: survival outcomes.

**Trial**	**Treatment arms**	**Overall survival**			**Progression-free survival or TTP**		
		
		**N**	**Median (mo)**	**Statistical comparison**	**N**	**Median (mo)**	**Statistical comparison**
Tannock, 2004 [64]	docetaxel q 3 wks prednisone	335	18.9	HR = 0.76 (95% CI, 0.62–0.94), p = 0.009	NR		
	docetaxel q wk prednisone	334	17.4	HR = 0.91 (95% CI, 0.75–1.11) p = 0.36			
	Mitoxantrone prednisone	337	16.5	NA			
Petrylak, 2004 [63]	Docectaxel EMP	338	17.5	HR = 0.80 (95% CI, 0.67–0.97) p = 0.02	324	6.3	HR = 0.73 (95% CI, 0.63–0.86) p < 0.001 (TPP)
	Mitoxantrone prednisone	336	15.6		324	3.2	
Abratt, 2004 [62]	Vinorelbine hydrocortisone ± AGM	206	14.7	p = 0.95	206	3.7	HR = 0.71 p = 0.055 (unadjusted) p = 0.005 (adjusted)*
	hydrocortisone ± AGM	208	15.2		208	2.8	
Berry, 2001 [84]	paclitaxel EMP	166	15.1	p = 0.11	166	NR	p = 0.08
	Paclitaxel		12.9				
Hudes, 1999 [61]	vinblastine EMP	95	11.9	p = 0.08	98	3.7	p < 0.0004† (TTP)
	Vinblastine	98	9.2		95	2.2	
Iversen, 1997 [65]	EMP	61	9.4	p = 0.09	60	2.2	
	Placebo	68	6.1		67	5.0	
Johansson, 1991 [66]	EMP	51	NR	p = 0.23	51	NR	p = 0.28
	MPA	51			51		
De Kernion, 1988 [67]	EMP	102	NR	p = NS	102	NR	p = NS
	Flutamide	101			101		
Murphy, 1979 [68]	EMP prednimustine	54	9.3	p = NS	NR		
	prednimustine	62	9.0				

*Adjusted for age, baseline hemoglobin, performance status, and alkaline phosphatase, and number of prior hormonal manipulations; †based on one-sided significance testing.

Abbreviations: AGM – aminoglutethimide; CI – confidence interval; EMP – estramustine phosphate; HR – hazard ratio; mo – months; MPA – medroxyprogesterone acetate; N – number; NA – not applicable; NR – not reported; NS – non-significant; q – every; TTP – time-to-progression; wk(s) – week(s).

**Table 3:** Antimicrotubule trials: PSA and tumor response.

**Trial**	**Treatment arms**	PSA response*			**Tumor response**		
		
		**N**	**Response rate %**	**Statistical comparison**	**N**	**Objective response rate %**	**Statistical comparison**
Tannock, 2004 [64]	docetaxel q 3 wks prednisone	291	45	p < 0.001	141	12	p = 0.1
	docetaxel q wk prednisone	291	48	p < 0.001	134	8	p = 0.6
	Mitoxantrone prednisone	300	32	NA	137	7	NA
Petrylak, 2004 [63]	Docetaxel EMP	309	50	p < 0.001	103	17	p < 0.30
	Mitoxantrone prednisone	303	27		93	11	
Abratt, 2004 [62]	Vinorelbine hydrocortisone ± AGM	206	30.1	p < 0.01	68	5.9(PR)	NR
	hydrocortisone ± AGM	208	19.2		74	0	
Berry, 2001 [84]	Paclitaxel EMP	166	48	p < 0.01	NR		
	Paclitaxel		25				
Hudes, 1999 [61]	vinblastine EMP	87	25.2	p < 0.0001	30	20 (PR)	p = 0.13
	Vinblastine	94	3.2		33	6 (PR)	
Iversen, 1997 [65]	EMP	43	37.2	p = 0.001	NR		
	Placebo	51	2.0				
Johansson, 1991 [66]	EMP	NR			NR		
	MPA						
De Kernion, 1988 [67]	EMP	NR			102	0	p = NS
	Flutamide				101	1.0 (PR)	
Murphy, 1979 [68]	EMP prednimustine	NR			54	1.9 (PR)	p = NS
	prednimustine				62	0	

*PSA response was defined as = 50 decrease in PSA compared with baseline.

Abbreviations: AGM – aminoglutethimide; EMP – estramustine phosphate; MPA – medroxyprogesterone acetate; N – number; NA – not applicable; NR – not reported; NS – non-significant; PR – partial response; PSA – prostate-specific-antigen; q – every; wk(s) – week(s).

**Table 4:** Mitoxantrone and anthracycline trials.

**Trial**	**N randomized/evaluable**	**Treatment arms (dose) and schedule**	**Duration**
Berry, 2002 [69]	120/119	mitoxantrone (12 mg/m^2^) iv q 3 wks prednisone (5 mg) po twice daily	6 cycles
		prednisone (5 mg) po twice daily	
Kantoff, 1999 [70]	242/242	mitoxantrone (14 mg/m^2^) iv q 3 wks hydrocortisone (40 mg) po daily (two divided doses)	hydrocortisone to progression or treatment failure
		hydrocortisone (40 mg) po daily (two divided doses)	
Tannock, 1996 [71]	161/161	mitoxantrone (12 mg/m^2^) iv q 3 wks prednisone (5 mg) po twice daily	mitoxantrone to dose of 140 mg/m^2^, continuing on prednisone
		prednisone (5 mg) po twice daily	
Weissbach, 1998 [88]	NR/175	epirubicin (25 mg/m^2^) iv q mo	NR
		EMP (560 mg) daily	
		mitomycin C (10 mg/m^2^) iv q mo	
Anderström, 1995 [72]	149/145	epirubicin (20 mg/m^2^) iv q wk MPA (500 mg) po twice daily	epirubicin to dose of 1000 mg/m^2^, MPA to progression
		EMP (12 mg/kg) po daily (two divided doses)	
Laurie, 1992* [73]	145/142	In combination: 5-FU (600 mg/m^2^) iv q 4–5 wks doxorubicin (30 mg/m^2^) iv q 4–5 wks mitomycin-C (10 mg/m^2^) iv q 4–5 wks†	to progression
		In sequence: 5-FU (500 mg/m^2^) iv q 5 wk doxorubicin (50 mg/m^2^) iv q 3–4 wk mitomycin-C (12.5 mg/m^2^) iv q 4 wk	
Saxman, 1992 [74]	103/103	cyclophosphamide (500mg/m^2^)‡ iv q 3 wks doxorubicin (50mg/m^2^)‡ iv q 3 wks methotrexate (40mg/m^2^)‡ iv q 3 wks	to progression, doxorubicin not to exceed dose of 450mg/m^2^
		cyclophosphamide (1000 mg/m^2^)§iv q 3 wks	
Murphy, 1988 [75]	180/152	doxorubicin (50 mg/m^2^) iv q 3 wks cyclophosphamide (500 mg/m^2^) iv q 3 wks	to progression
		cisplatin (50 mg/m^2^) iv q 3 wks 5-FU (500 mg/m^2^) iv q 3 wks cyclophosphamide (500 mg/m^2^) iv q 3 wks	
		methotrexate (100 mg/m^2^) iv (2 divided doses) q 2 wks	
Stephens, 1984 [76]	158/137	doxorubicin (40 mg/m^2^)¶iv q 3 wks cyclophosphamide (200 mg/m^2^)¶iv q 3 wks	doxorubicin to dose of 450 mg/m^2^, continuing on cyclophosphamide or hydroxyurea to progression
		hydroxyurea (3600 mg/m^2^) po twice q wk	

*This trial was terminated early due to declining patient accrual ; †after three courses, mitomycin-C was only given with every other course; ‡patients who had received prior radiation therapy were give cyclophosphamide, doxorubicin, and methotrexate at doses of 400 mg/m^2^, 40 mg/m^2^, and 32 mg/m^2^, respectively; §patients who had received prior radiation therapy were given cyclophosphamide at a dose of 800 mg/m^2^; ¶patients older than 65 years and with prior bone irradiation, and marrow invasion with tumor were deemed poor risk and were randomized to a reduced dose of doxorubicin (20 mg/m^2^) and cyclophosphamide (100 mg/m^2^).

Abbreviations: 5-FU – 5- fluorouracil; EMP – estramustine phosphate; iv – intravenous; m^2 ^- meters squared; mg – milligrams; mo – month; MPA – medroxyprogesterone; N – number; po – per oral; q – every; wk(s) – week(s)

**Table 5:** Mitoxantrone and anthracycline trials: survival outcomes.

**Trial**	**Treatment arms**	**Overall survival**			**Progression-free survival or TTP**		
		
		**N**	**Median (mo)**	**Statistical comparison**	**N**	**Median (mo)**	**Statistical comparison**
Berry, 2002 [69]	mitoxantrone prednisone	56	23	p = 0.48	56	8.1	p = 0.018 (TTP)
	prednisone	63	19		63	4.1	
Kantoff, 1999 [70]	mitoxantrone	119	12.3	p = 0.77	119	3.7	p = 0.02 (TTP)
	hydrocortisone	123	12.6		123	2.3	
Tannock, 1996 [71]	mitoxantrone prednisone	80	NR	p = 0.27	NR		
	prednisone	81					
Weissbach, 1998 [88]	epirubicin	61	NR by treatment group		61	NR by treatment group	"TTF was longer with mitomycin C vs. EMP (p = 0.037); and vs. epirubicin (p = 0.039)
	EMP	54			54		
	mitomycin C	60			60		
Anderström, 1995 [72]	epiribucin MPA	73	11.5	p = NS	73	7.6	p = 0.013 (TTP)
	EMP	72	9.5		72	4.3	
Laurie, 1992 [73]	5-FU doxorubicin mitomycin C (combined)	70	8.7	p = 0.025	NR		
	5-FU doxorubicin mitomycin C (sequential)	72	7.1				
Saxman, 1992 [74]	cyclophosphamide doxorubicin methotrexate	26 high PS 24 low PS	9.5 6	p = 0.93 p = 0.51 p = 0.7 (unstratified)	50	6.2*	p = 0.07 (TTP)
	cyclophosphamide	26 high PS 27 low PS	9 5		53	4.4*	
Murphy, 1988 [75]	doxorubicin cyclophosphamide	54	NR	p = NS	54	NR	p = NS
	cisplatin 5-FU cyclophosphamide	46			46		
	methotrexate	52			52		
Stephens, 1984 [76]	doxorubicin cyclophosphamide	68	6.8	p = NS	NR		
	hydroxyurea	69	7				

*Median values include only patients with partial response or stable disease (n was not reported).

Abbreviations: 5-FU – 5- fluorouracil; EMP – estramustine phosphate; mo – months; MPA – medroxyprogesterone; N – number; NR – not reported; NS – non-significant; PS – performance status; TTF – time-to-treatment failure; TTP – time-to-progression; vs – versus.

**Table 6:** Mitoxantrone and anthracycline trials: PSA and tumor response.

**Trial**	**Treatment arms**	PSA response*			**Tumor response**		
		
		**N**	**Response rate %**	**Statistical comparison**	**N**	**Objective response rate %**	**Statistical comparison**
Berry, 2002 [69]	mitoxantrone prednisone	56	48†	p = 0.007	8	25 (PR)	p = NR
	prednisone	63	24†		9	22 (PR)	
Kantoff, 1999 [70]	mitoxantrone hydrocortisone	96	18.7	p = 0.41	116	7 (PR)	p = 0.38
	hydrocortisone	91	14.3		118	4 (PR)	
Tannock, 1996 [71]	mitoxantrone prednisone	57	33	p = 0.11	NR		
	prednisone	54	22				
Weissbach, 1998 [88]	mitomycin C	NR			60	22	NR
	epirubicin				61	11	
	EMP				55	9	
Anderström, 1995 [72]	epiribucin MPA	NR			NR		
	EMP						
Laurie, 1992 [73]	5-FU doxorubicin mitomycin-C (combined)	NR			70	14	NR
	5-FU doxorubicin mitomycin-C (sequential)				72	18	
Saxman, 1992 [74]	cyclophosphamide doxorubicin methotrexate	NR			16	18.8 (PR)	NR
	cyclophosphamide				16	6 (PR)	
Murphy, 1988 [75]	doxorubicin cyclophosphamide	NR			54	1 (PR)	p = NS
	cisplatin 5-FU cyclophosphamide				46	0	
	methotrexate				52	0	
Stephens, 1984 [76]	doxorubicin cyclophosphamide	NR			19	32	p = 0.05
	hydroxyurea				24	4	

*PSA response was defined as e50% decrease in PSA compared with baseline; †PSA response with stabilization or improvement of performance status for at least 2 weeks.

Abbreviations: 5-FU – 5-fluorouracil; EMP – estramustine phosphate; N – number; NR – not reported; NS – non-significant; PR – partial response; PSA – prostate-specific antigen.

**Table 7:** Other chemotherapy trials.

**Trial**	**N randomized/Evaluable**	**Treatments (dose) and schedule**	**Duration**
Weissbach, 1998 [88]	NR/175	epirubicin (25 mg/m^2^) iv q mo	NR
		EMP (560 mg) daily	
		mitomycin C (10 mg/m^2^) iv q mo	
Newling, 1993 [77]	171/161	mitomycin-C (15 mg/m^2^) iv q 6 wks	to progression
		EMP (560 to 700 mg)* po daily	
Murphy, 1988 [75]	180/152	doxorubicin (50 mg/m^2^) iv q 3 wks cyclophosphamide (500 mg/m^2^) iv q 3 wks	to progression
		cisplatin (50 mg/m^2^) iv q 3 wks 5-FU (500 mg/m^2^) iv q 3 wks cyclophosphamide (500 mg/m^2^) iv q 3 wks	
		methotrexate (100 mg/m^2^) iv (2 divided doses) q 2 wks	
Loening, 1983 [78]	189/158	methotrexate (100 mg/m^2^) iv (two divided doses) q wk	12 wks
		cisplatin (60 mg/m^2^) iv (d1,4,21,24), then once monthly	
		EMP (600 mg/m^2^) po daily (3 divided doses)	

*Dose of estramustine escalated to 700 mg if 560 mg dose was tolerated for two weeks.

Abbreviations: 5-FU – 5- fluorouracil; d – day; EMP – estramustine phosphate; iv – intravenous; m^2 ^- meters squared; mg – milligrams; mo – month; N – number; NR – not reported; po – per oral; q – every; wk(s) – week (s).

**Table 8:** Non-cytotoxic trials.

**Trial**	**N randomized/evaluable**	**Treatments (dose) and schedule**	**Duration**
Carducci, 2004 [85]	809/809	atrasentan po (10 mg)	NR
		placebo	
Carducci, 2003 [79]	288/288	atrasentan (2.5 mg) po daily	to progression
		atrasentan (10 mg) po daily	
		placebo	
Small, 2003 [86]	127/127	APC8015 iv q 2 wks × 3	to progression
		placebo	
Small, 2002 [81]	390/390	suramin (3.192 mg/m^2^)* iv	3 cycles (12 weeks)
		suramin (5.320 mg/m^2^)* iv	
		suramin (7.661 mg/m^2^)* iv	
Ahmann, 2001 [87]	553/406†	prinomastat (5 mg) po twice daily mitoxantrone (12 mg/m^2^) iv q 3 wks prednisone (5 mg) po twice daily	NR
		prinomastat (10 mg) po twice daily mitoxantrone (12 mg/m^2^) iv q 3 wks prednisone (5 mg) po twice daily	
		mitoxantrone (12 mg/m^2^) iv q 3 wks prednisone (5 mg) po twice daily placebo	
Small, 2000 [82]	460/458	suramin d1: 1000 mg/m^2 ^2-hr iv d2-5: 400 mg/m^2^, 300 mg/m^2^, 250 mg/m^2^, and 200 mg/m^2 ^iv, respectively d8,11,15,19: 275 mg/m^2 ^iv for 2 wks d22,29,36,43,50,57,64,71,78: 275 mg/m^2 ^iv wks 4–12 hydrocortisone (40 mg) po daily	to progression or unacceptable toxicity
		hydrocortisone po daily placebo	
Debruyne, 1998 [83]	321/321	liarozole (300 mg) twice daily	to progression or unacceptable toxicity
		CPA (100 mg) twice daily	

*Doses of suramin decreased over 10 weeks; all patients received hydrocortisone at a dose of 25 mg orally each morning and 15 mg orally each evening; †interim results available for 406/553 patients.

Abbreviations: CPA – cyproterone acetate; d – day; DES – diethylstilbestrol diphosphate; g – grams; hr – hour; iv – intravenous; m^2 ^- meters squared; mg – milligram; N – number; NR – not reported; po – per oral; q – every; wk(s) – week(s); × – times.

**Table 9:** Non-cytotoxic trials: survival outcomes.

**Trial**	**Treatment arms**	**Overall survival**			**Progression-free survival or TTP or FFS**		
		
		**N**	**Median (mo)**	**Statistical comparison**	**N**	**Median (mo)**	**Statistical comparison**
Carducci, 2004 [85]	Atrasentan	NR			408	NR	HR for TTP = 1.14 (95% CI, 0.98–1.34) p = 0.091
	placebo				401		
Carducci, 2003 [79]	atrasentan 10 mg	NR			89	6.5	p = 0.13 (TTP)
	atrasentan 2.5 mg				95	6.4	p = 0.29 (TTP)
	placebo				104	4.9	NA
Small, 2003 [86]	APC8015	NR			82	NR	HR for TTP = 1.39 (95% CI, 0.95–2.04) p = 0.085
	placebo				45		
Small, 2002 [81]	suramin (3.192 g/m^2^)	128	16	p = 0.49	128	NR	p = NS
	suramin (5.320 g/m^2^)	124	14		124		
	suramin (7.661 g/m^2^)	120	13		120		
Ahmann, 2001 [87]	prinomastat (5 mg) mitoxantrone prednisone	134	15.1	p = NS	134	6	p = NS
	prinomastat (10 mg) mitoxantrone prednisone	134	14.7		134	4.7	
	mitoxantrone prednisone placebo	138	14.8		138	6	
Small, 2000 [82]	suramin hydrocortisone	228	10.2	p = NS	228	NR	RR for TTP = 1.51 (95% CI, 1.22–1.85) p = 0.0003
	placebo hydrocortisone	230	10		230		
Debruyne, 1998 [83]	liarozole	160	10.3	p = 0.52 HR = 0.74* (95% CI, 0.56–0.99) p = 0.039	160	4.9	p = NS
	CPA	161	10.3		161	4.6	

*Adjusted for performance status, hemoglobin, baseline PSA, alkaline phosphatase, and duration of response.

Abbreviations: CI – confidence interval; CPA – cyproterone acetate; DES – diethylstilbestrol; FFS – failure-free survival; HR – hazard ratio; m^2 ^- meters squared; mg – milligrams; mo – months; N – number; NA – not applicable; NR – not reported; NS – non-significant; RR – relative risk; TTP – time-to-progression.

**Table 10:** Non-cytotoxic trials: PSA and tumor response.

**Trial**	**Treatment arms**	PSA response*			**Tumor response**		
		
		**N**	**Response rate %, unless otherwise specified**	**Statistical comparison**	**N**	**Objective response rate %**	**Statistical comparison**
Carducci, 2004 [85]	Atrasentan	408	"smaller mean ↑ with atrasentan vs. placebo"	p = 0.025	NR		
	placebo	401					
Carducci, 2003 [79]	atrasentan 10 mg	median time-to-PSA progression:			NR		
		89	5.5 mo	p = 0.002			
	atrasentan 2.5 mg	95	5 mo	p = 0.055			
	placebo	104	2.5 mo	NA			
Small, 2003 [86]	APC8015	82	4.9	NR	NR		
	placebo	45	0				
Small, 2002 [81]	suramin (3.192 g/m^2^)	128	24	p = 0.08 (test for trend)	128	9	p = 0.104 (test for trend)
	suramin (5.320 g/m^2^)	124	28		124	7	
	suramin (7.661 g/m^2^)	120	34		120	15	
Ahmann, 2001 [87]	prinomastat (5 mg) mitoxantrone prednisone	134	17†	p = NS	NR		
	prinomastat (10 mg) mitoxantrone prednisone	134	18†				
	mitoxantrone prednisone placebo	138	14†				
Small, 2000 [82]	suramin hydrocortisone	228	33	p = 0.01	76	4 (PR)	NR
	placebo hydrocortisone	230	16		80	0	
Debruyne, 1998 [83]	liarozole	160	20	p < 0.001	NR		
	CPA	161	4				

*PSA response was defined as = 50% decrease in PSA compared with baseline; †75% reduction in PSA for 3 weeks.

Abbreviations: CPA – cyproterone acetate; DES – diethylstilbestrol; g – grams; mg – milligrams; mo – months; N – number; NA – not applicable; NR – not reported; NS – non-significant; PR – partial response; PSA – prostate-specific-antigen; vs. – versus.
