# Supplementary material for: Non-hormonal systemic therapy in men with hormone-refractory prostate cancer and metastases: a systematic review from the Cancer Care Ontario Program in Evidence-based Care's Genitourinary Cancer Disease Site Group
Source: BMC Cancer. 2006 May 2;6:112. doi: 10.1186/1471-2407-6-112 (PMC1550253; doi:10.1186/1471-2407-6-112)
Supplement: Additional file 1 — Appendix of ineligible randomized trialsThe file is a word document and describes the randomized trials that were excluded from the systematic review because they did not meet the eligibility criteria. [file 1471-2407-6-112-S1.doc]

Appendix: Ineligible randomized trials.

| **Trial** | **N** | **Treatment arms** |
| --- | --- | --- |
| Scott, 1977 [8] | 110 | 5-FU |
|  |  | cyclophosphamide |
|  |  | standard treatment |
| Eagen, 1976 [9] | 26 | doxorubicin |
|  |  | 5-FU + cyclophosphamide |
| Kvols, 1977 [10] | 16 | melphalan |
|  |  | ICRF-159 |
|  |  | hydroxyurea |
| Murphy, 1977 [11] | 125 | EMP |
|  |  | streptozotocin |
|  |  | standard treatment |
| Tejada, 1977 [12] | 18 | 5-FU |
|  |  | CCNU |
| Chlebowski, 1978 [13] | 27 | cyclophosphamide |
|  |  | cyclophosphamide + 5-FU + doxorubicin |
| Schmidt, 1979 [14] | 129 | cyclophosphamide |
|  |  | DTIC |
|  |  | procarbazine |
| Loening, 1981 [15] | 123 | hydroxyurea |
|  |  | cyclophosphamide |
|  |  | CCNU |
| Muss, 1981 [16] | 32 | cyclophosphamide |
|  |  | cyclophosphamide + MTX + 5-FU |
| Smalley, 1981 [17] | 71 | cyclophosphamide + doxorubicin + 5-FU |
|  |  | 5-FU |
| Soloway, 1981 [18] | 90 | EMP |
|  |  | EMP + vincristine |
|  |  | vincristine |
| Herr, 1982 [19] | 40 | cyclophosphamide + MTX + 5-FU |
|  |  | CCNU |
| DeWys, 1983 [20] | 99 | doxorubicin |
|  |  | 5-FU |
| Soloway, 1983 [21] | 124 | EMP |
|  |  | cisplatin |
|  |  | EMP + cisplatin |
| Kasimis, 1985 [22] | 30 | cyclophosphamide |
|  |  | 5-FU + doxorubicin + mitomycin C |
| Page, 1985 [23] | 47 | doxorubicin + lomustine |
|  |  | cyclophosphamide + 5-FU |
| Torti, 1985 [24] | 37 | doxorubicin + cisplatin |
|  |  | doxorubicin |
| Benson, 1989 [25] | 60  (subgroup) | EMP |
|  |  | DES |
| Graham, 1986 [26] | 58 | mephalan + MTX + vincristine + 5-FU + prednisone |
|  |  | cyclophosphamide + 5-FU + MTX + prednisone |
| Akaza, 1988 [27] | 26 | EMP + peplomycin + doxorubicin |
|  |  | 5-FU + peplomycin + doxorubicin |
| Kitahara, 1988 [28] | 22 | ifosfamide + 5-FU + cisplatin |
|  |  | ifosfamide |
| Manni, 1988 [29] | 85 | androgen priming  [AGM + hcort + cyclophosphamide + 5-FU + doxorubicin, then MTX, fluorouracil] |
|  |  | no priming  [AGM + hcort + cyclophosphamide + 5-FU + doxorubicin, then MTX, fluorouracil] |
| McLeod, 1988 [30] | 86 | megace |
|  |  | megace + DES |
|  |  | stilphostrol |
| Papdopoulos, 1989 [31] | 30 | cisplatin + epirubicin |
|  |  | cisplatin + epirubicin + factor AF2 |
| Ruff, 1989 [32] | 57 | MPA |
|  |  | chlorambucil |
|  |  | cyclophosphamide + doxorubicin + 5-FU |
| Shafik, 1990 [33] | 36 | MTX per annum |
|  |  | MTX iv |
| Tveter, 1990 [34] | 79 | EMP |
|  |  | epirubicin + MPA |
|  |  | epirubicin + placebo |
| Elomaa, 1991 [35] | 41 | EMP |
|  |  | epirubicin (low dose) |
| Rangel, 1992 [36] | 52 | doxorubicin + prednisone |
|  |  | prednisone |
| Francini, 1993 [37] | 72 | epirubicin |
|  |  | doxorubicin |
| Daliani, 1995 [38] | 51 | 5-FU + interferon-alpha |
|  |  | 5-FU |
| Breul, 1997 [39] | 49 | 5-FU |
|  |  | 5-FU + folinic acid |
| Brune, 1998 [40] | 82 | pirarubicin |
|  |  | AGM + hcort |
| van Andel, 2000 [41] | 28 | epirubicin + MPA |
|  |  | epirubicin |
| Dahut, 2002 [42] | 75 | docetaxel + thalidomide |
|  |  | docetaxel |
| Figg, 2001[43] | 63 | thalidomide (low dose, 200mg) |
|  |  | thalidomide (high dose, 1200mg) |
| Millikan, 2001 [44] | 89 | ketoconazole + doxorubicin |
|  |  | ketoconazole |
| Tombal, 2003 [46] | 61 | irofulven + prednisone |
|  |  | irofulven |
| Hervonen, 2002 [45] | 30 | ifosfamide (24hr infusion d1) |
|  |  | ifosfamide (3hr infusion d1-4) |
| Oudard, 2002 [48] | 130 | docetaxel (70mg/m^2^ d2) + EMP + prednisone |
|  |  | docetaxel (35mg/m^2^ d2, d8) + EMP + prednisone |
|  |  | mitoxantrone + prednisone |
| Tolcher, 2002 [47] | 30 | antisense oligonucleotide ISIS 3521 |
|  |  | antisense oligonucleotide ISIS 5132 |
| Droz, 2003 [49] | 42 | oxaliplatin + 5-FU |
|  |  | oxaliplatin |
| Millikan, 2003 [50] | 71 | ketoconazole + doxorubicin + vinblastine + EMP |
|  |  | paclitaxel + EMP + etoposide |
| Salimichokami, 2003 [51] | 55 | docetaxel + thalidomide |
|  |  | docetaxel |
| Sternberg, 2003 [52] | 50 | satraplatin + prednisone |
|  |  | prednisone |
| van Andel, 2003 [53] | 79 | epirubicin weekly |
|  |  | epirubicin q 4 wks |
| Albrecht, 2004 [54] | 90 | EMP + vinblastine |
|  |  | EMP |
| Dimopoulos, 2004 [55] | 38 | EMP + etoposide |
|  |  | LHRH analogue + somatostatin analogue (lanreotide) + dexamethasone |
| Birch, 2004 [56] | 62 | docetaxel (36mg/m^2^) + EMP |
|  |  | docetaxel (70mg/m^2^) + EMP |
| Eymard, 2004 [57] | 92 | docetaxel + EMP |
|  |  | docetaxel |
| Kelly, 2004 [58] | 43 | epothilone B analogue BMS-247550 + EMP |
|  |  | epothilone B analogue BMS-247550 |
| Lara, 2004 [59] | 80 | MMPI BMS-275291 (1200mg) |
|  |  | MMPI BMS-275291 (2400mg) |
| Millikan, 2004 [60] | 150 | ketoconazole + doxorubicin alternating with vinblastine + EMP |
|  |  | cyclophosphamide + vincristine + dexamethasone |
|  |  | paclitaxel + EMP + etoposide |
|  |  | paclitaxel + EMP + carboplatin |

Abbreviations: 5-FU – 5-fluorouracil; AGM - aminoglutethimide; CCNU – lomustine; d – day; DES – diethylstilbestrol; DTIC – dacarbazine; EMP – estramustine phosphate; hcort – hydrocortisone; hr – hour; iv – intravenously; LHRH – luteinizing hormone-releasing hormone; m^2^ – meters squared; mg – milligrams; MMPI – matrix metalloproteinase inhibitor; MPA – medroxyprogresterone; MTX – methotrexate; N – number; q – every; wks – weeks.
